# Sepsis-like histoplasmosis in a kidney transplant patient

**DOI:** 10.1590/1678-4685-JBN-3767

**Published:** 2018-04-23

**Authors:** Ana Raquel Fernandes, Laila Almeida Viana, Juliana Busato Mansur, Mariana de Moraes Françoso, Daniel Wagner de Castro Lima Santos, Helio Tedesco Silva, José Osmar Medina Pestana

**Affiliations:** 1 Serviço de Nefrologia do Centro Hospitalar de Setúbal, Portugal.; 2 Hospital do Rim, São Paulo, SP, Brasil.

**Keywords:** kidney transplant, immunosuppression, sepsis, opportunistic infections, transplante de rim, imunossupressão, sepse, infecções oportunistas

## Abstract

Histoplasmosis is a fungus infection that mainly affects immunosuppressed patients. The authors present a case of a kidney transplant recipient who developed sepsis-like histoplasmosis, na atypical but severe manifestation of the disease. The fungus was found in blood and in a skin biopsy, and the treatment with liposomal amphotericin resulted in hepatotoxicity.

## Resumo

Histoplasmose é uma infecção fúngica que afeta principalmente pacientes imunossuprimidos. Os autores apresentam um caso de uma receptora de transplante de rim que desenvolveu histoplasmose disseminada, uma manifestação atípica, mas grave da doença. O fungo foi encontrado no sangue e na biópsia cutânea, e o tratamento com anfotericina lipossomal resultou em hepatotoxicidade.

## Introduction

Kidney transplant patients are prone to opportunistic infections due to immunosuppression. Histoplasma is a systemic mycosis caused by *Histoplasma capsulatum* var. *capsulatum* or *Histoplasma capsulatum* var. *durboisii* that mostly affects immunocompromised hosts*.* The latter has been identified in Africa and Europe, whereas *Histoplasma capsulatum* var. *capsulatum* is endemic in North and Latin America^1^. This disease was rarely diagnosed in Brazil before AIDS (acquired immune deficiency syndrome), but in the 1980's and 1990's, patients with this syndrome presented diverse forms of histoplasmosis, especially the disseminated type^2^. Renal transplant recipients and patients with hematological malignancies are the most commonly affected by this illness^2^, transmitted via feces of birds and bats dispersed on the ground^3^.

Post-transplant histoplasmosis is a potentially lethal event^4^ with an incidence of less than 0.5%^5^^-^ 8. The development of the disease is host-dependent^9^ and its presentation can be focal, systemic, or disseminated. There are three routes of infection: (a) inhalation of soil aerosol contaminated with bird or bat excreta, (b) endogenous reactivation, and (c) transmission from donor-infected tissue^10^. Severe disease can present as sepsis syndrome with hypotension, disseminated intravascular coagulation, renal failure, and acute respiratory distress. In most cases, death is the spontaneous evolution of the severe form^11^^-^ 12.

Here, we report the case of a patient with an atypical and severe sepsis-like histoplasmosis.

## Case report

**Figure 1:**
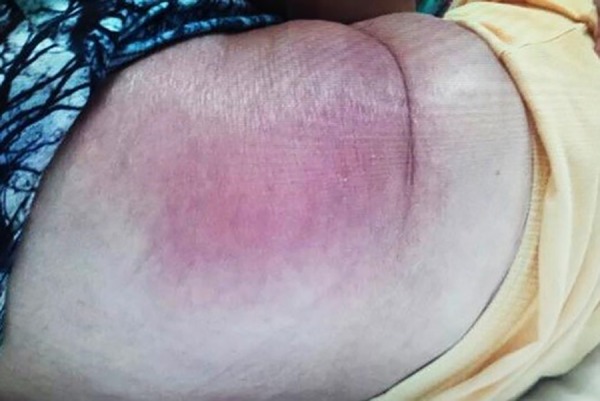
Rash on the back of the patient on admission of the patient.

A sixty-four-year-old renal transplant recipient woman presented at the hospital six years after transplantation. She had had a history of fever, hypotension, and asthenia with 36 hours of evolution associated with seven kilograms of weight loss in three months. Physical examination revealed white spots in the mouth and a rash on the back (Figure 1). The patient had anemia (Hb 8.4 g/dL, Ht 23.0%), leukopenia (2.3×10^9^/L), C reactive protein (10.8 mg/dL) and ferritin (13.253 ng/mL) above the normal range, and acute graft dysfunction (Cr 3.0 > 4.0mg/dL). Liver function was not compromised (albumin 3.7g/dL, aspartate aminotransferase 32 U/L, alanine aminotransferase 29 U/L). Her immunosuppression regimen at admission was prednisone (5 mg/day) and azathioprine (2 mg/Kg/day), and the basal serum creatinine (Cr) value was 3.0 mg/dL.

On the day of her admission, hemocultures were performed to search for fungus and mycobacterium. Polymerase chain reaction (PCR) for CMV and Epstein Barr virus (EBV), latex agglutination test for cryptococcus, and serology for *Histoplasma capsulatum* and *Trypanosoma cruzi* were also done. From that day, an empiric treatment with vancomycin, meropenem, and ganciclovir was started aiming to treat a possible bacterial infection or a viral opportunistic one.

CMV antigenemia was negative but PCR unveiled 546 copies of the virus. A decision was made to perform a biopsy of the cutaneous lesion and cultivate fungi and mycobacteria. For further investigation, image tests such as a thoraco-abdominal computerized tomography scan (CT) and an echocardiogram were done, which did not reveal any abnormality. A myelogram showed erythroid hyperplasia and an upper digestive endoscopy showed an ulcerative esophagitis with intranuclear inclusions and some esophageal candidiasis lesions. Fluconazole was associated with the current antimicrobial and antiviral regimen (vancomycin, meropenem and gancyclovir).

The clinical condition of the patient declined, she had melena and mucocutaneous bleeding with hemodynamic instability, which evolved to disseminated intravascular coagulation (DIC). We empirically started liposomal amphotericin B to cover histoplasmosis and a four-drug treatment for tuberculosis. The patient did not improve and became unstable, needing mechanical ventilation and vasopressor support. She had acute hepatitis due to liposomal amphotericin B hepatotoxicity and died 7 days after starting the treatment for histoplasmosis.

The diagnosis of sepsis-like histoplasmosis was done after laboratory results obtained post-mortem. *Histoplasma capsulatum* was found in blood cultures and serology by double immunodiffusion and immunoblotting (band H and M). The microbiology of the skin biopsy also isolated *H. capsulatum*.

## Discussion

Certain infections in kidney transplant recipients may have an atypical symptomatic presentation due to immunosuppression^13^. The clinical frame of histoplasmosis is not specific since 50% percent of patients develop respiratory symptoms and 75% disseminate histoplasmosis^14^, characterized by the involvement of at least two organs^5^ Twenty-five to 60% of patients have hepatosplenomegaly. Septic shock is frequent^15^ and some patients present a syndrome characterized by hypotension, respiratory insufficiency, renal and hepatic failure and coagulopathy - the sepsis-like syndrome, which occurs in 10-20% of the patients with AIDS^16^, though the frequency in kidney transplant patients is unknown. Our patient presented a sepsis-like histoplasmosis (skin and hematological involvement culminating in a septic shock) without respiratory illness.

We could have tried to integrate the lesions found in the esophagus as another symptom of the disease. The gastrointestinal (GI) tract lesions in histoplasmosis occur predominantly in the ileocecal region, but the upper GI tract can also be involved in the form of oropharyngeal ulceration. The histologic pattern should be one of the following 1) no visible gross abnormalities but presence of infected macrophages in the lamina propria; 2) collection of infected macrophages presenting as pseudopolyps or plaques; 3) ulceration with tissue necrosis; and 4) localized inflammation leading to stricture formation^17^. Because none of these patterns was seen in our patient, we did not consider an involvement of the GI system. Moreover, almost all immunosuppressive medications are associated with some forms of GI complications^18^^-^ 21, the majority of which fall into one of the general categories: infections (bacterial, viral, fungal, or parasitic), mucosal injury and ulceration, biliary tract diseases, diverticular disease, pancreatitis, and malignancy^22^.

Although histoplasmosis is a rare clinical suspicion, it is highly important in these cases. A retrospective study of 61 patients with disseminated histoplasmosis reported a mortality rate of 31% in immunocompromised patients and 17% in immunocompetent patients^23^. The diagnosis is based on the detection of the fungus in body fluids (sputum, blood, cerebrospinal fluid) or tissues (histopathology), by biological culture and serological assays^24^. In our case, evidence was found in blood and skin; there was no fungus growth in bone marrow and we did not screen the respiratory system, as there was no pulmonary manifestation of the disease. Patients with severe disseminated infection should be treated initially with amphotericin B at a dosage of 0.7 to 1 mg/kg daily or a lipid formulation of amphotericin B at a dosage of 3 to 5 mg/kg daily, owing to their reduced toxicity, for 12-18 months^1^. Unfortunately, hepatotoxicity is common as verified in our patient.

There are few cases similar to this one in the literature and most are with HIV patients. A similar case was described by Vaidya et al.^11^, although their patient had an exuberant skin lesion and no severe hematologic evolvement.

## Conclusion

Sepsis-like histoplasmosis is usually severe and fulminant. Our patient did not show any improvement with the therapeutics and manifested significant adverse effects that led to a change of treatment strategy. Transplanted patients are vulnerable to several rare diseases that can be fatal; sepsis-like histoplasmosis is one of them.
